# Saddle-Reset for Robust Parameter Estimation and Identifiability Analysis of Nonlinear Mixed Effects Models

**DOI:** 10.1208/s12248-020-00471-y

**Published:** 2020-07-02

**Authors:** Henrik Bjugård Nyberg, Andrew C. Hooker, Robert J. Bauer, Yasunori Aoki

**Affiliations:** 1 Department of Pharmaceutical Biosciences, Uppsala University, Uppsala, Sweden; 2 Pharmacometrics R&D, ICON CLINICAL RESEARCH LLC, Gaithersburg, Maryland USA; 3 Present Address: National Institute of Informatics, Tokyo, Japan

**Keywords:** estimation methods, NLME, parameter estimation, pharmacometrics, practical identifiability

## Abstract

Parameter estimation of a nonlinear model based on maximizing the likelihood using gradient-based numerical optimization methods can often fail due to premature termination of the optimization algorithm. One reason for such failure is that these numerical optimization methods cannot distinguish between the minimum, maximum, and a saddle point; hence, the parameters found by these optimization algorithms can possibly be in any of these three stationary points on the likelihood surface. We have found that for maximization of the likelihood for nonlinear mixed effects models used in pharmaceutical development, the optimization algorithm Broyden–Fletcher–Goldfarb–Shanno (BFGS) often terminates in saddle points, and we propose an algorithm, saddle-reset, to avoid the termination at saddle points, based on the second partial derivative test. In this algorithm, we use the approximated Hessian matrix at the point where BFGS terminates, perturb the point in the direction of the eigenvector associated with the lowest eigenvalue, and restart the BFGS algorithm. We have implemented this algorithm in industry standard software for nonlinear mixed effects modeling (NONMEM, version 7.4 and up) and showed that it can be used to avoid termination of parameter estimation at saddle points, as well as unveil practical parameter non-identifiability. We demonstrate this using four published pharmacometric models and two models specifically designed to be practically non-identifiable.

## INTRODUCTION

Inaccurately estimated parameter values can introduce bias and inflate uncertainty, which in turn will influence any decisions supported by modeling and simulation results. There exist many parameter estimation methods for nonlinear mixed effects models (1–11). In this paper, we focus on maximum likelihood-based parameter estimation algorithms where the likelihood is approximated either by the first-order approximation (first order, FO; first-order conditional estimate, FOCE) or second-order approximation (Laplace approximation) and then maximized using a gradient-based optimization algorithm. More specifically, we focus our investigation on minimization of the approximated − 2log likelihood (objective value function, OFV) using the Broyden–Fletcher–Goldfarb–Shanno (BFGS) algorithm (12) implementation in NONMEM (13), a software package for population pharmacometric modeling that is commonly used for regulatory submission.

**Fig. 1:**
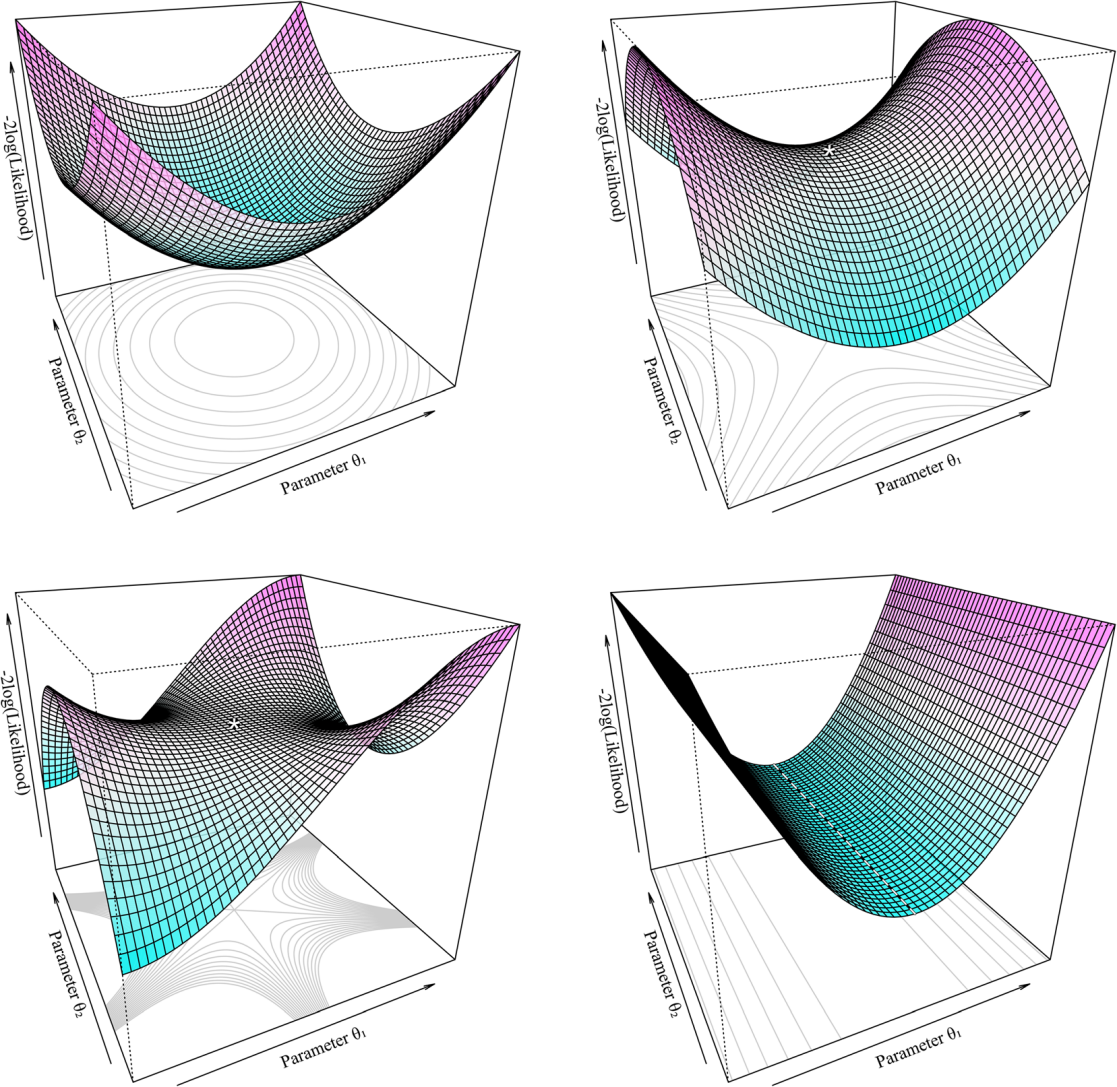
Examples of the stationary point where $\nabla \mathrm{OFV}=\overrightarrow{0}$ for the case of two parameter model (i.e., *p* = 2). Top left: a minimum on the surface, where the curvature is positive in all directions. Top right: a saddle point, marked *, where the curvature is negative in one direction around a point, but positive in the other. Bottom left: a so-called monkey saddle, a degenerate saddle point with reversing curvature (inflection) around a point. Bottom right: a region of non-identifiability, where the curvature is zero in one direction, and all values of *θ*_1_ produce the same, lowest OFV value along a vector

The OFV forms a surface in (*p* + 1)-dimensional space, where *p* is the number of estimated parameters. BFGS moves iteratively to points across this surface in search of a stationary point, a point where the gradient of the objective function is a zero vector. This can be thought of as solving a system of nonlinear equations $\nabla \mathrm{OFV}=\overrightarrow{0}$, where the Hessian matrix (or its approximation) determines the direction the point is moved at each iteration. As can be seen in Fig. 1, for the case of two estimated parameters (i.e., *p* = 2), the stationary point is a necessary, but not sufficient condition for the point to be at a minimum. See Appendix I for further mathematical background.

In this paper, we show that the maximum likelihood estimation of nonlinear mixed effects models using BFGS can terminate prematurely at saddle points. Then we propose an algorithm, saddle-reset, to move the parameter away from such non-minimum stationary points. We implemented the proposed algorithm in NONMEM (version 7.4 and above), and using this implementation, we show that the proposed algorithm helps us find more accurate maximum likelihood estimates. We also show that the proposed algorithm can unveil non-identifiability of a parameter for the case where the parameter is not locally practically identifiable. The NONMEM implementation is used by setting SADDLE_RESET=*N*, where *N* is the number of consecutive user-requested repetitions of the algorithm.

Several approaches to the saddle point problem have been suggested, for example, modified Newton methods or methods using stochastic gradients (14,15). The proposed algorithm is based on the second derivative test, similar to an approach first used by Fiacco and McCormick (16,17), and uses the Hessian of the OFV to derive the optimal direction of the perturbation.

## METHODS

### Saddle-Reset Algorithm

Let *f* be a map from model parameter vector ***θ*** to − 2log(likelihood). We aim to find the maximum likelihood parameter which is defined as $\hat{\boldsymbol{\theta}}={argmin}_{\theta}\left(f\left(\boldsymbol{\theta} \right)\right)$. We consider $\overset{\sim }{\boldsymbol{\theta}}$, a numerical approximation of $\hat{\boldsymbol{\theta}}$, using a local search algorithm for solving a system of nonlinear equations (e.g., Quasi-Newton methods, gradient-based methods, BFGS) by solving ∇*f*(***θ***) = 0. We denote this operation of applying the algorithm to numerically approximate local minima of *f*(***θ***), by an operator *F*, where *F* takes a nonlinear function *f* and the initial guess of the minima ***θ***_init_ as the inputs. The operator *F* outputs $\overset{\sim }{\boldsymbol{\theta}}$ the numerical approximation of the local minima of the nonlinear function *f* near the initial guess ***θ***_init_. We denote this operation as $\overset{\sim }{\boldsymbol{\theta}}=F\left(f\left(\bullet \right),{\boldsymbol{\theta}}_{\mathrm{init}}\right)$.

We assume that the algorithm finds a stationary point of a function near a given initial guess ***θ***_init_, i.e.:

1 $$\overset{\sim }{\boldsymbol{\theta}}=F\left(f\left(\bullet \right),{\boldsymbol{\theta}}_{\mathrm{init}}\right)$$

such that

2 $$\nabla f\left(\overset{\sim }{\boldsymbol{\theta}}\right)=0$$

3 $$f\left(\overset{\sim }{\boldsymbol{\theta}}\right)\le f\left({\boldsymbol{\theta}}_{\mathrm{init}}\right).$$

The stationary point can be classified using a Hessian matrix, and we denote the Hessian matrix of − 2log(likelihood) as the *R*-matrix, i.e.:

4 $${r}_{ij}\left(\boldsymbol{\theta} \right)=\frac{\partial^2}{\partial {\theta}_i\partial {\theta}_j}f\left(\boldsymbol{\theta} \right)$$

where *r*_*ij*_ is the element of the matrix R at the *i*th row and *j*th column. Note that if *f* is nonlinear, this matrix depends on ***θ*** so we will denote the *R*-matrix that is evaluated at ***θ*** as *R*(***θ***). Lastly, we denote *p* as the number of parameters in the parameter vector ***θ***; hence, *R*(***θ***) is a *p* × *p* matrix. The algorithm can use either the computed Hessian matrix after the end of the BFGS search (*R*-matrix) or the BFGS Hessian approximation from the last iteration of the search as a substitute.

The algorithm consists of the following 8 steps:

1. Estimate the maximum likelihood parameters by finding a stationary point near an initial guess ***θ***_init_ using a gradient-based local search algorithm and denote it as $\overset{\sim }{\boldsymbol{\theta}}$ (see Eq. (1)).
2. If an element in the gradient vector cannot be computed at $\overset{\sim }{\boldsymbol{\theta}}$ (e.g., the numerical integration of the model ODE for that derivative component fails), then reset the associated parameter values in $\overset{\sim }{\boldsymbol{\theta}}$ to those from *θ*_init_ (initial parameters at start of estimation) and proceed to step 6 with this new ${\boldsymbol{\theta}}_{\mathrm{init}}^{\mathrm{new}}$.
3. Compute the Hessian, or acquire the BFGS Hessian approximation, at the stationary point $\overset{\sim }{\boldsymbol{\theta}}$.
4. Find the lowest eigenvalue *λ*_*l*_ and the associated unit eigenvector ***v***_*l*_ of the Hessian, i.e.:
  5 $${\lambda}_l{\boldsymbol{v}}_l=R\left(\overset{\sim }{\theta}\right){\boldsymbol{v}}_l$$
  6 $${\boldsymbol{v}}_l^T{\boldsymbol{v}}_l=1$$
5. Select new initial parameter values by a second-order Taylor series approximation along *v*_*l*_ to find an approximate change in OFV of 1, i.e.:
  7 $${\theta}_{\mathrm{init}}^{\mathrm{new}}=\overset{\sim }{\boldsymbol{\theta}}+\sqrt{\frac{2}{\left|{\lambda}_l\right|}}{v}_l$$
  with a protection for cases where *λ*_*n*_ → 0 and step length would approach ∞, i.e.:
  8 $${\boldsymbol{\theta}}_{\mathrm{init}}^{\mathrm{new}}=\overset{\sim }{\boldsymbol{\theta}}+\min \left({\max}_i\left(\frac{1}{2}\left|\frac{{\overset{\sim }{\boldsymbol{\theta}}}_i}{v_{l,i}}\right|\right),\sqrt{\frac{2}{\left|{\lambda}_l\right|}}\right){v}_l$$
  Further justification for Eqs. (7) and (8) is shown in Eq. (11–17) in Appendix II.
6. Resume parameter estimation to find a stationary point near new initial guess ${\boldsymbol{\theta}}_{\mathrm{init}}^{\mathrm{new}}$ using the gradient-based local search algorithm, i.e.:
  9 $${\overset{\sim }{\boldsymbol{\theta}}}^{\mathrm{new}}=F\left(f\left(\bullet \right),{\boldsymbol{\theta}}_{\mathrm{init}}^{\mathrm{new}}\right)$$
7. Check if the *N* user-requested saddle-resets have been performed. If reset steps remain, return to step 2, replacing $\overset{\sim }{\boldsymbol{\theta}}$ with ${\overset{\sim }{\boldsymbol{\theta}}}^{\mathrm{new}}.$
8. Conclude the parameter estimation at ${\overset{\sim }{\boldsymbol{\theta}}}^{\mathrm{new}}$.

#### A Note on Step 2

In the case that there are numerical problems with the evaluation of a gradient element, then the BFGS implementation in NONMEM sets that gradient element to zero, the eigenvalue becomes zero, and the associated eigenvector becomes a unit vector along the axis of the parameter with numerical issues. If this vector is selected and used in steps 3–5, then the parameter with the numerical problem would be changed without any relation to the curvature of the − 2log likelihood surface (see Eq. (8)). In this situation, the parameter with the numerical problem is instead set to its initial value.

### NONMEM Implementation

We have implemented saddle-reset in NONMEM 7.4. It is enabled by specifying the option SADDLE_RESET = *N* on the $ESTIMATION record, where *N* is the number of resets to perform before concluding parameter estimation. The option is applicable only when BFGS is used to maximize the likelihood approximated by FO, FOCE, or Laplace approximations.

In order to reduce runtime, NONMEM by default uses the approximation of the Hessian matrix from the last iteration of the BFGS method in step 3 of the algorithm. As this matrix is already computed at the last iteration of BFGS, using this matrix instead of computing the Hessian saves computational cost. However, note that the BFGS approximation of the Hessian is constructed to be positive definite and hence cannot be used for the second derivative test (i.e., it cannot be used to classify the stationary point). If the SADDLE_HESS = 1 option is specified, NONMEM will instead compute the Hessian (*R*-matrix), Eq. (4), in step 3 of the algorithm.

### Numerical Experiments

To demonstrate the utility of the proposed algorithm in realistic and practical settings, we have obtained four published nonlinear mixed effects models in pharmacometrics with the original datasets. These four examples are chosen from a wide range of pharmacokinetics (models for the time-course change of drug concentration) and pharmacokinetic-pharmacodynamic models (models of a biomarker or endpoint that is driven by the pharmacokinetics model). In addition, to demonstrate the algorithm’s usefulness for detecting practical non-identifiability, we have created two nonlinear mixed effects models with one simulated dataset each, so that one would be structurally non-identifiable and another would be practically non-identifiable. An overview of the selected models is presented in Table I. For details of the published models, we refer the reader to the original publications (18–21). For details of the non-identifiable models, see Appendix III.

**Table I:** Models Used for Numerical Experiments

Model	Reference	Model classification	Fixed effects	Random effects	Residual error	Number of subjects	Number of samples	Comment
A	Jönsson *et al.* (18)	Two-comp. PK	7	2	Additive	177	1196	Closed form
B	Bergmann *et al.* (19)	Two-comp. PK	10	3	Additive and proportional	93	274	Closed form
C	Wählby *et al.* (20)	Two-comp. PK, transit comp. power PD	7	4	Additive and proportional	47	530	ODEs
D	Grasela and Donn (21)	One-comp. PK,	3	3	Proportional	59	155	Closed form
E		Practically non-identifiable Emax model.	8	5	Additive and proportional	326	1803	ED_50_ and γ cannot both be estimated on sim. Data
F	Non-identifiable example from Aoki *et al.* (22)	Structurally non-identifiable two-comp. PK w/ fraction of dose data	4	3	Proportional	25	612	V1, Q, V2, and CL cannot all be estimated

*Comp.*, compartment; *DEs*, differential equations; *γ*, hill factor for Emax model; *ED*_*50*_, dose required for half effect; *V1*, volume of central compartment; *V2*, volume of peripheral compartment; *Q*, intercompartmental clearance; *CL*, clearance

Parameter estimation was performed on each model using 1000 sets of initial parameters generated uniformly and at random within, proportionally, 99% above and below the best-known parameter values for the identifiable models, or true parameter values used for simulation for the non-identifiable models, according to Eq. (10),

10 $${\boldsymbol{\theta}}_{\mathrm{init},k}\sim {\boldsymbol{\theta}}_{\mathrm{best}}+U\left({\boldsymbol{\theta}}_{\mathrm{best}}\ast 0.01,{\boldsymbol{\theta}}_{\mathrm{best}}\ast 1.99\right)$$

where ***θ***_init,*k*_ is the *k*th generated set of initial values, ***θ***_best_ is the best-known parameter value, and U(a,b) is a uniform random variable generated between *a* and *b*. This procedure was done using Perl speaks NONMEM (23) (PsN). Given that some of the parameters are off-diagonal elements of a variance-covariance matrix for random effects of the models, and the variance-covariance matrix needs to be positive definite, if the randomly generated initial parameter vector resulted in a non-positive definite variance-covariance matrix, then a replacement matrix was constructed from its eigendecomposition, replacing any negative eigenvalues with a small positive value (i.e., 10^−10^).

For the examples with original data (models A–D), we do not know the true parameter vector so we use the published parameter values as the best-known parameter values. Note that for all of these examples, throughout our rich numerical experiment (i.e., many thousands of parameter estimations using a wide range of initial estimates), we have not found any better parameter sets (higher likelihood) than those published. For models E and F, where simulated data is used, the parameters used for simulation were the best-known parameter values.

For each model, estimation of $\overset{\sim }{\boldsymbol{\theta}}$ was performed from each of the 1000 initial parameter values using the following methods:

- Default estimation: Gradient-based estimation performed using the method originally used in the published model.
- Random perturbation and re-estimation: Gradient-based estimation performed using the method originally used in the published model (the default estimation method, above), plus two subsequent estimations. One starting from the final parameter estimates of the default estimation, and one starting from a randomly selected ${\boldsymbol{\theta}}_{\mathrm{init}}^{\mathrm{new}}$ from a uniform distribution spanning, proportionally, 10% above and below each of the final estimates of the default estimation. The result with the lowest – 2log(likelihood) of the two estimations is then selected, regardless of NONMEM estimation status.
- Saddle-reset: Saddle-reset was tested with three different settings: (1) a single saddle-reset step using the BFGS Hessian approximation (SADDLE_RESET = 1), (2) three consecutive reset steps using the BFGS Hessian approximation (SADDLE_RESET = 3), and (3) a single saddle-reset step using the computed Hessian (SADDLE_RESET = 1 SADDLE_HESS = 1). Three saddle-resets were included in order to compare one saddle-reset and confirm whether one reset is sufficient.

“Estimation success” for identifiable models A–D was evaluated by if the estimation methods reached within 1 point above the minimum known – 2log(likelihood) for that model/data combination.

For the non-identifiable models (E and F), the methods were evaluated based on the change in maximum likelihood parameter estimates compared with default estimation, calculated as the difference divided by the true value. For identifiable models, the maximum likelihood estimate is a single value within numerical error. If a method can produce a changed parameter value with the same lowest known OFV, we consider that as having exposed local, practical non-identifiability of that parameter. A method that finds the same lowest known OFV with a larger change in the parameter value, translating to a wider distribution of delta values over the 1000 estimations in our experiment, is considered more successful, as this makes the non-identifiability more apparent.

## RESULTS

### Identifiable Models

**Fig. 2:**
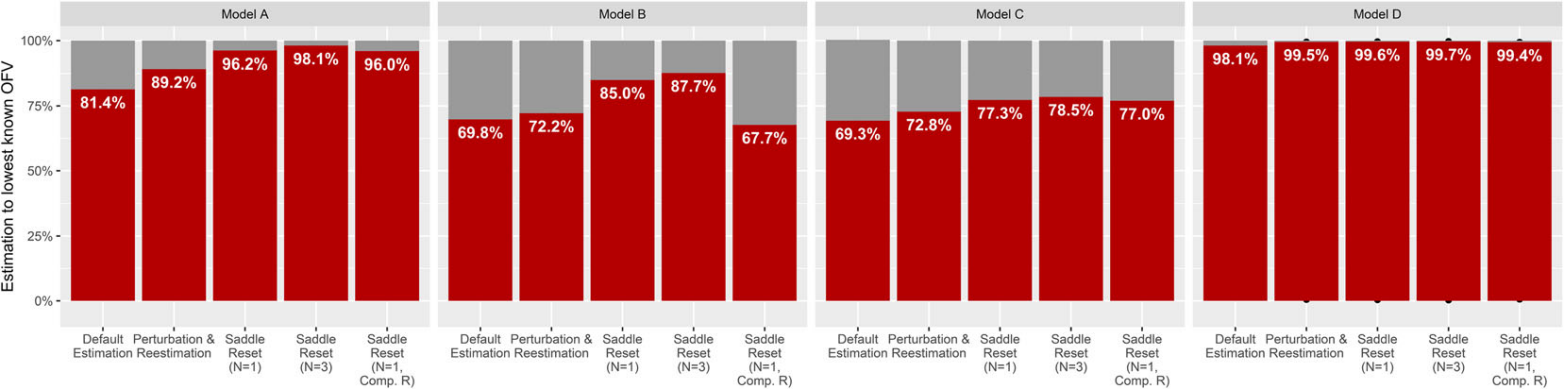
Success rate of default estimation, perturbation, and re-estimation, and saddle-reset (1 time, 3 times, and 1 time with computed R-matrix) for models A–D. Successful minimizations to within one point above the lowest known OFV are counted (OFV ≤ lowest known OFV + 1). Comp. R marks saddle-reset with computed R-matrix (SADDLE_HESS = 1)

The default method failed to find the lowest known OFV in a portion of estimations for all models. Compared with default estimation, all other methods had a higher portion of estimations that reached the lowest known – 2log(likelihood) in all models, with the exception of saddle-reset with computed R-matrix for model B, where many estimations crashed. Saddle-reset consistently outperformed random perturbation and re-estimation, with a larger portion of estimations reaching the lowest known − 2log(likelihood) for each tested model. The success rates for each examined identifiable model and method are shown in Fig. 2.

Using the default estimation method, maximum likelihood estimates were found to have terminated prematurely in saddle points for all identifiable models between 1.6 and 26.5% of the time, as categorized by the positive definiteness of the computed R-matrix, see Table II.

**Table II:** Final Status of the Default Estimation Method for the Identifiable Example Models. The Distinction Between Local Minima and Saddle Points Was Made by Calculating the R-Matrix at the Final Estimate and Evaluating Its Positive Definiteness. This Calculation Includes Numerical Approximation and the Classification Is Not Conclusive

	Estimated to best-known minimum OFV	Estimated to local minimum	Estimated to saddle point	Crashed estimations
Model A	814	13	171	2
Model B	698	25	265	12
Model C	693	53	126	128
Model D	981	0	16	3

**Fig. 3:**
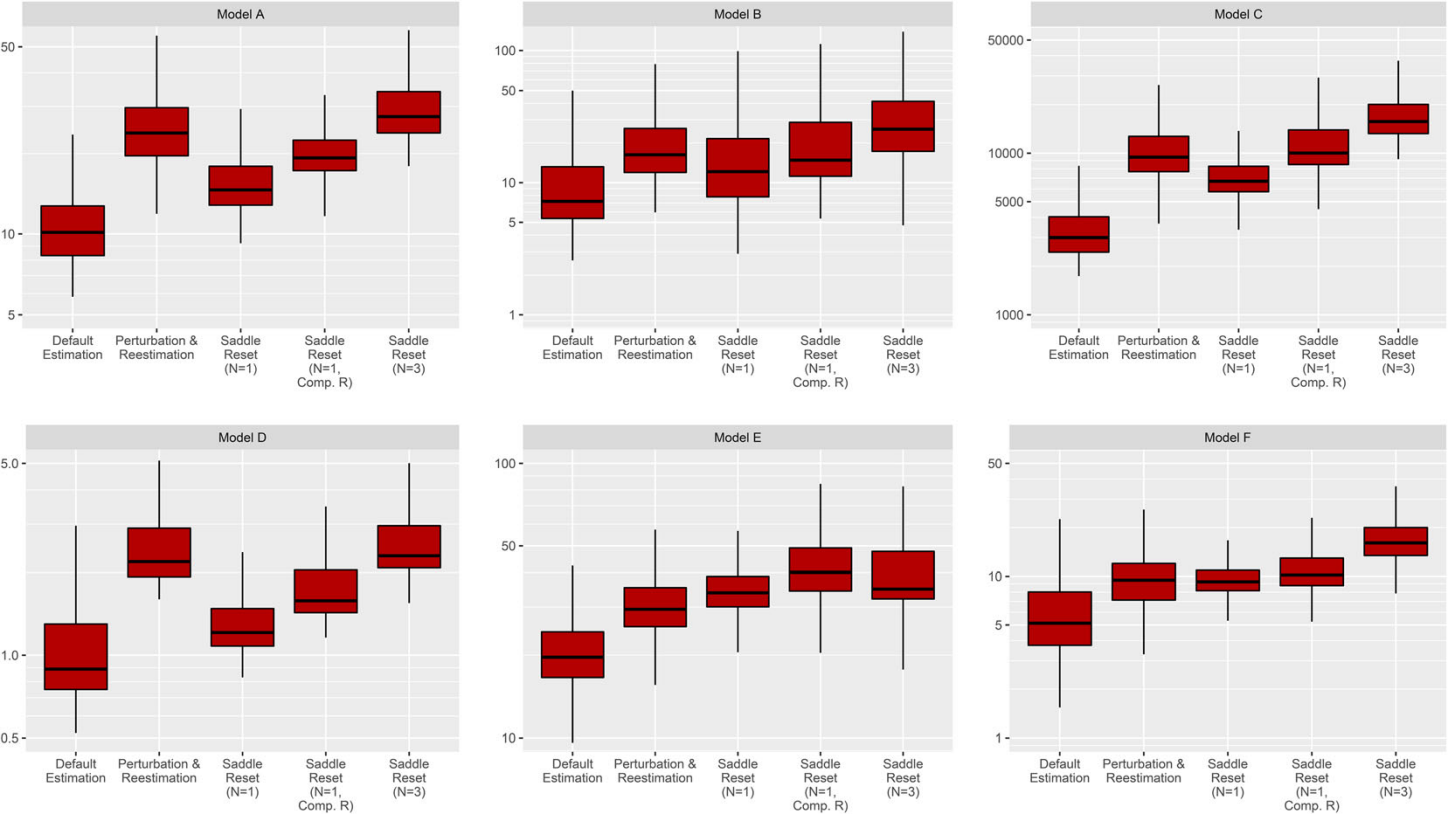
Boxplots of estimation time in seconds for the default estimation, random perturbation, and re-estimation, and saddle-reset for all models. Note that the *y*-axes have different logarithmic scales for the different models. Comp. R marks saddle-reset with computed R-matrix (SADDLE_HESS = 1)

Boxplots of runtimes for the different methods and models are presented in Fig. 3. For the identifiable models A–D, performing a single saddle-reset increased estimation time by a median 65% over default estimation. Perturbation and re-estimation increased runtime in the same estimations by a median of 118%.

Performing multiple saddle-reset steps in a single estimation had only a small positive effect on estimation success rate. Employing three saddle-reset steps (SADDLE_RESET = 3) instead of one (SADDLE_RESET = 1) only improved success rate by 1.4% on average across models A–D, while having a major impact on runtime as shown in Fig. 3.

Using saddle-reset with computed R-matrix for identifiable models gave marginally worse estimation results than a single saddle-reset step with the BFGS-approximated Hessian for models A, C, and D. For model B, the method was unstable, with 157 of the 1000 estimations producing no OFV, compared with 11 and 13, respectively, for default estimation and one saddle-reset step.

### Non-Identifiable Models

**Fig. 4:**
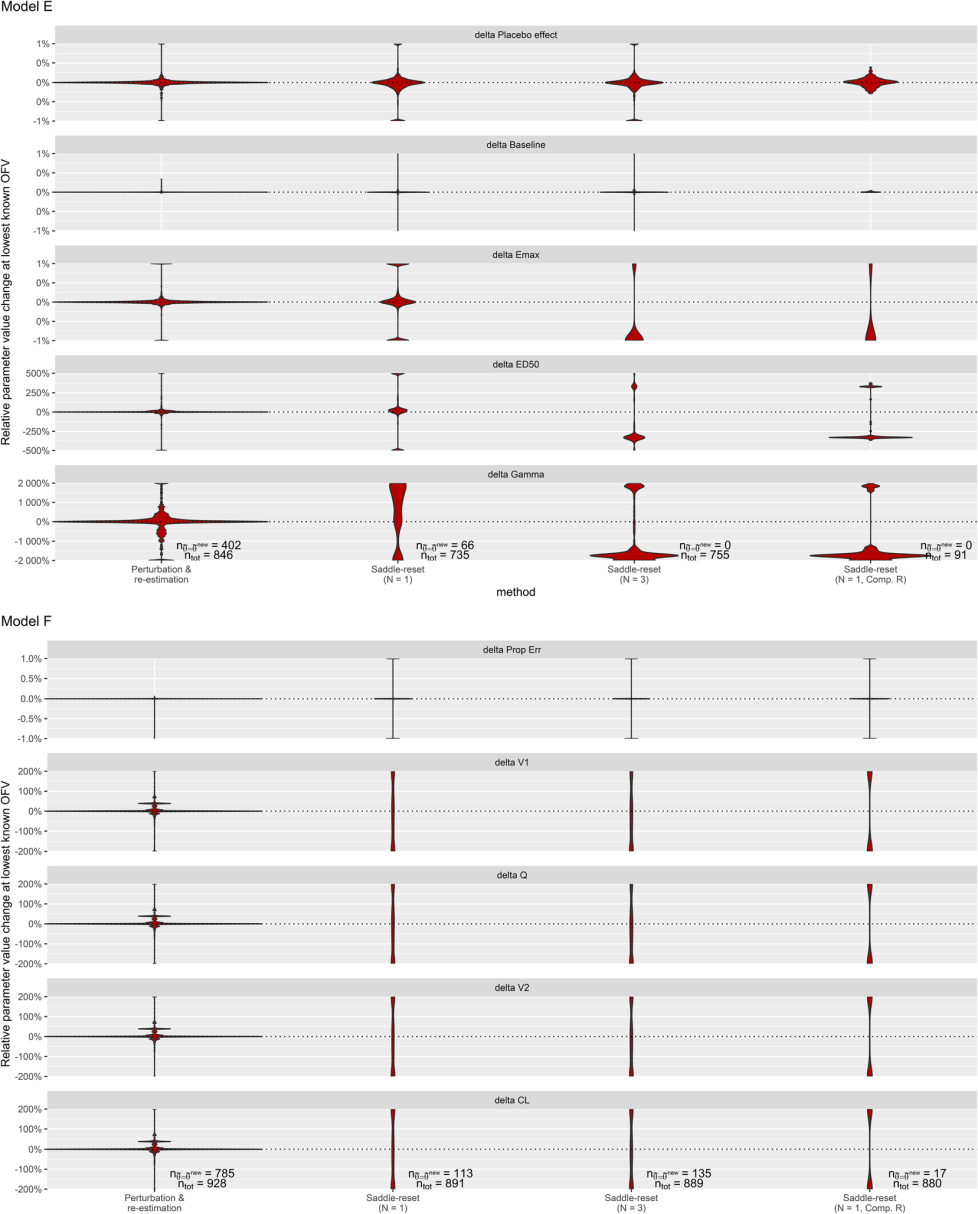
Violin plots displaying change in selected fixed effects parameter values between the respective method and default estimation, relative to true values, delta values in percent, for the non-identifiable models E (top) and F (bottom), at their respective lowest − 2log(likelihood). The four methods compared are, in order from the left, perturbation and re-estimation, one saddle-reset step, three saddle-reset steps, and one saddle-reset step with computed R-matrix. A wider distribution and separation from zero indicates better performance in exposing the non-identifiability. Using a computed R-matrix produces parameter values that are vastly different from the default estimation, clearly indicating non-identifiability. Some parameters remain identifiable, such as baseline in model E and proportional error in model F. The total number of estimations that reached the lowest known OFV (n_tot_), and the number of estimations that produced the same parameter estimates in default estimation and in the respective method (n_θ̃=θ̃_^new^), is shown in the bottom panel for each method in each model. A lower n_tot_ indicates that estimations crashed or did not reach the lowest OFV. A lower n_θ̃=θ̃_^new^ means that more estimations unveiled non-identifiability

Different parameter estimates producing the same − 2log(likelihood) are evidence of non-identifiable parameters. In model E, the parameters ED50 and Gamma cannot be simultaneously identified, and in model F, the parameters’ volume of the central compartment (V1), clearance (CL), volume of the peripheral compartment (V2), and intercompartmental clearance (Q) cannot be simultaneously identified. Figure 4 shows violin plots of the change in parameter estimates between the default estimation and each of the compared methods, for estimations of models E and F that reach within 1 point of their lowest known − 2log(likelihood) for the compared methods. The saddle-reset algorithm produced changed parameter values at a higher rate than perturbation and re-estimation. For both models E and F, saddle-reset identified a wide range of parameter values for the non-identifiable or non-estimable parameters at the minimum known − 2log(likelihood), translating into a wide distribution of absolute delta parameter values.

Three consecutive saddle-reset steps provided very similar results to one saddle-reset, although delta ED50 and delta Gamma in model E are completely separated from zero by three saddle-reset steps, meaning that the non-identifiability is unveiled in every estimation that reaches the lowest known OFV.

Using saddle-reset with computed R-matrix greatly improved the results for model F, but the method was unstable for model E. Out of the 850 model E estimations that reached the lowest known OFV in default estimation, only 91 did so after saddle-reset with computed R-matrix.

Runtime with a single saddle-reset step was on par with perturbation and re-estimation for both non-identifiable examples (as seen in Fig. 3). Performing saddle-reset with a computed R-matrix or performing three consecutive saddle-resets came at a very small additional computational cost for these two models.

## DISCUSSION

This work has presented saddle-reset, an algorithm to improve the BFGS optimization method used to obtain maximum likelihood parameters in pharmacometric models, and to simultaneously check for local practical non-identifiability. The proposed algorithm was more likely to find accurate maximum likelihood parameters compared with conventional methods and with random perturbation methods. In addition, based on the implementation we have tested, a single saddle-reset required less computational time than the random perturbation method.

Both saddle-reset and random perturbation successfully unveiled local non-identifiability by producing changed parameter values at the lowest known OFV, with a single saddle-reset step providing more distinctly different values of the non-identifiable parameters in a larger portion of estimations for both examples. One saddle-reset step was similar in performance to random perturbation and re-estimation for model E, while being significantly better for model F. This discrepancy in the relative performance is likely due to two things: the number of parameters involved in the non-identifiability, with model F having four non-identifiable parameters compared with two parameters for model E, and the required precision in step direction. The structurally non-identifiable example can be exposed by evaluating parameter values along many different directions around the estimated parameter values, while the practically non-identifiable example requires a more precise step direction. These differences between the examples may also help explain why using a computed Hessian (i.e., SADDLE_HESS = 1) was of great benefit for the structurally non-identifiable model F, but was very unstable for the practically non-identifiable model E.

The use of the approximate Hessian matrix from the last iteration of the BFGS algorithm did not affect the algorithm’s ability to surpass saddle points in the identifiable examples, and it was more stable for models B and E. However, using the numerically computed Hessian (i.e., setting SADDLE_RESET = 1 and SADDLE_HESS = 1) greatly improved the algorithm’s performance in unveiling non-identifiable parameters for the cases where estimation was successful, producing vastly different parameter values at the same, lowest known OFV. Although the finite difference scheme for the Hessian incurs additional computational cost, resulting in longer runtime in all examples, it may be more appropriate to use when identifiability issues are indicated or suspected.

At a saddle point, there are two possible directions along the selected eigenvector, positive and negative. Preliminary experiments using both directions did not significantly improve performance (results not shown). This came as a surprise to us since our intuition was that a saddle point would, at least in some sense, be a divider between two areas of the surface. The explanation for the results is likely that this intuitive understanding underestimated the flexibility of these systems.

This work has certain limitations. The saddle-reset algorithm is unlikely to be effective for unveiling global non-identifiability for cases that are locally identifiable, such as flip-flop kinetics. Similarly, the method is not designed to surpass local minima, although we would like to note that what are colloquially referred to as local minima may often actually be saddle points, as the classification results in Table II indicate. The implementation of a multi-start algorithm (24) such as libensemble (25) may be a possible extension for the presented research to overcome these challenges. We have also not evaluated the impact of different step length (OFV change of 1 point) or different eigenvector directions in the saddle-reset step. Future improvements could add a layer to the algorithm to, for example, test multiple different eigenvectors or step lengths, or to select the best result of several consecutive saddle-reset steps. As presented here, saddle-reset is a single sequential process just like BFGS. Lastly, we assume the likelihood surface to be twice continuously differentiable, and that the Hessian therefore exists, but this is not always the case for nonlinear mixed effects models in pharmacometrics. However, with the approximation of the hessian in the BFGS algorithm, some of the effects of this assumption can be overcome.

## CONCLUSION

Saddle-reset is an efficient and easy-to-use algorithm for exposing and avoiding saddle points and local practical identifiability issues in parameter estimation. We recommend using one saddle-reset step (implemented as SADDLE_RESET = 1 in NONMEM) when performing maximum likelihood-based parameter estimation by maximizing likelihood using gradient-based numerical optimization algorithms (e.g., FO, FOCE, LAPLACE).

## Appendix I. Mathematical background

### Interpretation of Hessian and the Shape of the OFV Surface

The Hessian of the objective function, also known as the *R*-matrix (see Eq. (4)), describes the curvature of the OFV surface. The geometrical feature of the OFV surface along the eigenvector ***v***_*i*_ of the R-matrix can be classified by the associated eigenvalues *λ*_*i*_ as follows:

- *λ*_*i*_ = 0 flat
- *λ*_*i*_ < 0 concave (maximum)
- *λ*_*i*_ > 0 convex (minimum)

In addition, the stationary point (the point where the gradient is the zero vector) can be classified using these eigenvalues as follows:

- If all eigenvalues of the *R*-matrix are positive, then the point is a local minimum.
- If the *R*-matrix has positive and negative eigenvalues, then the point is a saddle point.
- If at least one eigenvalue is zero, then this means that the point is either:
  - a saddle point with inflection such as the monkey saddle, or
  - that the surface does not change along the direction of that eigenvector.

The classification of different stationary points according to the above rules is trivial if the Hessian is evaluated exactly at the location of the stationary point; however, one should keep in mind that the Hessian obtained using computational algorithms is subject to rounding error. For example, a low surface curvature with one small positive eigenvalue (a local minimum) can be computationally difficult to separate from a negative (saddle point) or zero (non-identifiable parameter) eigenvalue. Many methods also use approximations of the Hessian that are biased or restrained. For example, BFGS uses an approximation of the Hessian that is made to be positive definite, and thus cannot be used to classify the stationary point.

### Saddle Points

A saddle point is a stationary point on a surface, i.e., a point where the gradient is zero, around which there is at least one direction with decreasing function value, and at least one of increasing function value. On objective function surfaces, this means that there are better parameter values that can be found with a local step in the right direction. See the top right and bottom left panels of Fig. 1 for two examples of saddle points.

Saddle-reset can surpass saddle points by taking a step along the lowest curvature and thus reach a lower objective function value from which to resume estimation.

### Non-Identifiability

It is important to differentiate between structural identifiability, where all parameters can be identified with infinite data available, and practical identifiability, sometimes called estimability or deterministic identifiability, where all parameters can be identified with the available data. Locally structurally non-identifiable models are, by their nature, also practically non-identifiable and can be exposed as locally practically non-identifiable using the same local methods. Structural non-identifiability has been studied extensively (26–28) and is not a common problem in pharmacometrics as shown for example in excellent analyses by Janzen *et al.* (29) and Shivva *et al.* (30). Practical non-identifiability, on the other hand, is a prominent problem (31).

In a flat region of the objective function, there are multiple sets of parameter values that yield the same objective function value. If there exists two or more separate such sets for a model/data combination, then there is no optimal value of at least one parameter, and the model is non-identifiable. This can have implications for modeling efforts that use the likelihood ratio test, since it defies Wilk’s theorem and thus hampers the assumption that likelihood ratios follow a *χ*^2^ distribution (32,33). The bottom right panel of Fig. 1 shows the simplest example of non-identifiability, where a change in value of one parameter has no impact on the − 2log(likelihood). This produces a line, rather than a point that, in this example, runs along one parameter axis. While such a line is not a stationary point, it may appear as such to a gradient-based search algorithm due to rounding errors or the search path.

Saddle-reset can expose non-identifiability by taking a step along the line of optimal values and thus showing the same − 2log(likelihood) for different parameter values before and after the saddle-reset step and re-initiated estimation.

## Appendix II. Justification for Eqs. (8) and (9)

Claim: $\left|f\left(\sqrt{\frac{2}{\left|{\lambda}_l\right|}}{v}_l+\overset{\sim }{\theta}\right)-f\left(\overset{\sim }{\theta}\right)\right|\approx 1$ for small $\sqrt{\frac{2}{\left|{\lambda}_l\right|}}$.

Proof: Consider the second-order Taylor series expansion of *f* at $\overset{\sim }{\theta }$, i.e.:

11 $$f\left(\overset{\sim }{\theta }+\Delta \theta \right)\approx f\left(\overset{\sim }{\theta}\right)+\Delta {\theta}^T\nabla f\left(\overset{\sim }{\theta}\right)+\frac{1}{2}\Delta {\theta}^TR\left(\overset{\sim }{\theta}\right)\Delta \theta$$

for small *∆θ*. We will now let $\Delta \theta =\sqrt{\frac{2}{\left|{\lambda}_l\right|}}{v}_n$ and assume that $\sqrt{\frac{2}{\left|{\lambda}_l\right|}}{v}_n$ is small, (e.g., *λ*_*l*_ ≠ 0):

12 $$f\left(\overset{\sim }{\theta }+\sqrt{\frac{2}{\left|{\lambda}_l\right|}}{v}_l\right)\approx f\left(\overset{\sim }{\theta}\right)+\sqrt{\frac{2}{\left|{\lambda}_l\right|}}{v}_l^{\mathrm{T}}\nabla f\left(\overset{\sim }{\theta}\right)+\frac{1}{2}\sqrt{\frac{2}{\left|{\lambda}_l\right|}}{v}_l^{\mathrm{T}}R\left(\overset{\sim }{\theta}\right)\sqrt{\frac{2}{\left|{\lambda}_l\right|}}$$

For small $\sqrt{\frac{2}{\left|{\lambda}_l\right|}}{v}_n$. Assuming $\overset{\sim }{\theta }$ is at a stationary point, i.e., $\nabla f\left(\overset{\sim }{\theta}\right)=0$ (cf. Eq. (2)), and some calculation, Eq. (12) can be simplified as follows:

13 $$f\left(\overset{\sim }{\theta }+\sqrt{\frac{2}{\left|{\lambda}_l\right|}}{v}_l\right)\approx f\left(\overset{\sim }{\theta}\right)+\frac{1}{\left|{\lambda}_l\right|}{v}_l^{\mathrm{T}}R\left(\overset{\sim }{\theta}\right){v}_l$$

14 $$=f\left(\overset{\sim }{\theta}\right)+\frac{1}{\left|{\lambda}_l\right|}{v}_l^{\mathrm{T}}{\lambda}_l{v}_l\kern1.25em \left(\mathrm{since}\ {v}_l\ \mathrm{is}\ \mathrm{an}\ \mathrm{eigenvector},\mathrm{cf}.6\right)$$

15 $$=f\left(\overset{\sim }{\theta}\right)+\frac{\lambda_l}{\left|{\lambda}_l\right|}\kern4.5em \left(\mathrm{since}\ {v}_l\ \mathrm{is}\ \mathrm{a}\ \mathrm{unit}\ \mathrm{vector},\mathrm{cf}.7\right)$$

By subtracting $f\left(\overset{\sim }{\theta}\right)$ from both sides of Eq. (15), we have the following:

16 $$f\left(\overset{\sim }{\theta }+\sqrt{\frac{2}{\left|{\lambda}_l\right|}}{v}_l\right)-f\left(\overset{\sim }{\theta}\right)\approx \frac{\lambda }{\left|{\lambda}_l\right|}$$

17 $$=\left\{\begin{array}{c}1\kern2.5em if\ {\lambda}_l>0\\ {}-1\kern1.75em if\ {\lambda}_l<0\end{array}\right.$$

The claim is proven.

## Appendix III. Detailed description of non-identifiable models

### Model E – Practically Non-Identifiable Emax Model

The model expresses a biomarker for an individual *i*, measured during visit *j* to a clinic (*y*_*i,j*_), as a function of fixed effects (*θ*) inter-individual random effects (*η*_*i*_), covariate effects (*β*_i_), dose (*D*), and additive and proportional residual error (*ε*_Add*,i,j*_, *ε*_Prop,*i,j*_).

18 $${\displaystyle \begin{array}{c}{y}_{i,j}=f\left(\theta, {\upeta}_i,{\beta}_i,D,{\varepsilon}_{i,j}\right)=\left({E}_{\mathrm{Baseline},i}+{E}_{\mathrm{Placebo},i}+{E}_{\mathrm{Drug},i,j}\right)\left(1+{\varepsilon}_{\mathrm{Prop},i,j}\right)+{\varepsilon}_{\mathrm{Add},i,j}\\ {}{\varepsilon}_{\mathrm{Prop},i,j}\sim N\left(0,{\sigma}^2\right),{\varepsilon}_{\mathrm{Add},i,j}\sim N\left(0,{\sigma}^2\right),\end{array}}$$

19 $${\displaystyle \begin{array}{c}{E}_{\mathrm{Baseline},i}={\theta}_{\mathrm{Baseline}}\ast {\beta}_{\mathrm{Sex},i}\ast \left(1+{\beta}_{\mathrm{Age}}\left({\mathrm{Age}}_i-45.16\right)+{\beta}_{FEV1}\ast \left( FEV{1}_{PN,i}-70\right)\right)\ast {e}^{\eta_{\mathrm{Baseline},i}}\\ {}{\eta}_{\mathrm{Baseline},i}\sim N\left(0,{\upomega}^2\right)\end{array}}$$

20 $${E}_{\mathrm{Placebo},i}={\theta}_{\mathrm{Placebo}}+{\eta}_{\mathrm{Placebo},i},\kern0.5em {\eta}_{\mathrm{Placebo},i}\sim N\left(0,{\upomega}^2\right)$$

21 $${E}_{\mathrm{Drug},i,j}=\left\{\begin{array}{c}0\kern8em if\ j<4\\ {}{E}_{\mathrm{max}}\frac{D_i^{\upgamma}}{D_i^{\upgamma}+{ED_{50}}^{\upgamma}}\kern1.75em if\ j\ge 4\end{array}\right.$$

The parameter values used for simulations, and as the center for the random perturbation before estimation, are shown in Table III.

**Table III:** Model E Pharmacodynamic Parameter Values Used for Simulation. For Estimation, the Random Perturbation Was Made Around These Values

Parameter	Typical value	ω (IIV, app. SD scale)	σ (residual error, app. SD scale)
E_Baseline_	2.55013	0.1	
E_Placebo_	0.0676556	0.1	
E_max_	0.137501		
ED_50_	10		
γ	0.6304		
β_Sex_			
Male	0.715994		
Female	1		
β_Age_	− 0.0116814		
β_FEV1_	0.0129253		
Additive error			0.1
Proportional error			0.1

*IIV*, inter-individual variability; *app. SD scale*, estimate of variability on approximate standard deviation scale; *E*_*Placebo*_, placebo effect; *E*_*Baseline*_, baseline effect; *E*_*max*_, maximal effect; *γ*, hill factor for Emax model; *ED*_*50*_, dose required for half effect; *β*_*Sex*_, sex effect on baseline; *β*_*Weight*_, weight effect on baseline; *β*_*Age*_, age effect on baseline; *β*_*FEV1*_, FEV1 effect on baseline; *FEV1*, forced expiratory volume in 1 s;

The study design has 326 individuals making a total of 1803 observation visits after receiving a dose of 0, 10, 40, or 400 units. Each individual makes six (*n* = 261), five (*n* = 18), four (*n* = 19), three (*n* = 15), or two (*n* = 13) observation visits. An example dataset for a single individual is shown in Table IV.

**Table IV:** Model E Example Data for One Individual

ID	Visit	Age	Sex	FEV1_PN_	Dose	*y*
2	2	41	2	67.7	40	3.18
2	3	41	2	67.7	40	3.01
2	4	41	2	67.7	40	2.723675
2	5	41	2	67.7	40	3.013675
2	6	41	2	67.7	40	2.443675
2	7	41	2	67.7	40	2.793675

### Model F - Structurally Non-Identifiable Two-Comp. PK with Fraction of Dose Data

The model expresses observed fraction of dose amount data in an individual *i* at time *t* (*y*_*i,t*_) as a function of fixed effects (*θ*) inter-individual random effects (*η*_*i*_), time (*t*), dose (*D*), and proportional residual error (*ε*_*i,t*_). The variables *u*_1_ and *u*_2_ denote the fractions of absorbed amount in compartment one and two, respectively, after an arbitrary bolus dose at time *t* = 0.

22 $${y}_{i,t}=f\left(\theta, {\eta}_i,t,{\varepsilon}_{i,t}\right)={u}_{1,i,t}\left(1+{\varepsilon}_{i,t}\right),\kern0.5em {\varepsilon}_{i,t}\sim N\left(0,{\sigma}^2\right)$$

23 $$\frac{d}{dt}{u}_{1,i}=-{CL}_i\frac{u_1}{V_1}-Q\frac{u_1}{V_1}+Q\frac{u_2}{V_2}$$

24 $$\frac{d}{dt}{u}_{2,i}=Q\frac{u_1}{V_1}-Q\frac{u_2}{V_2}$$

25 $${CL}_i={\theta}_{CL}\ast {e}^{\eta_{CL,i}},\kern0.75em \left[\genfrac{}{}{0pt}{}{\eta_{CL,i}}{\eta_{V_1,i}}\right]\sim {N}_2\left(\overrightarrow{0},\varOmega \right)$$

26 $${V}_{1,i}={\theta}_{V_1}\ast {e}^{\eta_{V_1,i}},\kern0.75em \left[\genfrac{}{}{0pt}{}{\eta_{CL,i}}{\eta_{V_1,i}}\right]\sim {N}_2\left(\overrightarrow{0},\varOmega \right)$$

27 $$Q={\theta}_Q$$

28 $${V}_2={\theta}_{V_2}$$

For proof of the non-identifiability, please see Aoki *et al.*, appendix section C.2.5 (22). The model was implemented using ADVAN3 TRANS4 in NONMEM.

The parameter values used for simulations, and as the center for the random perturbation before estimation, are shown in Table V.

**Table V:** Model F Pharmacokinetic Parameter Values Used for Simulation

Parameter	Typical value	ω (IIV, variance scale)	Σ (residual error, approx. SD scale)
CL	2.825120	0.211405	
CL – V1 IIV covariance		− 0.01629	
V1	4.189603	0.211405	
Q	15.32572		
V2	9.830136		
Proportional error			0.103916

*IIV*, inter-individual variability; *SD*, standard deviation; *CL*, clearance; *V1*, volume of the central compartment; *Q*, intercompartmental clearance; *V2*, volume of the peripheral compartment

The study design includes 612 observations of fraction of amount in 25 individuals after a dose of 100 units. An example dataset for a single individual is shown in Table VI.

**Table VI:** Model F Example Data for One Individual

ID	Time	*y*	Dose amount
1	0	0	100
1	1.6116	6.6235	0
1	2.2645	4.4116	0
1	3.3643	3.7568	0
1	4.1419	3.0119	0
1	5.9355	1.7335	0
1	7.2877	1.0309	0
1	8.9445	0.69203	0
1	9.1647	0.56351	0
1	9.7232	0.63195	0
1	11.258	0.32338	0
1	11.691	0.23928	0
1	11.759	0.32525	0
1	12.632	0.23712	0
1	14.506	0.11139	0
1	15.03	0.10224	0
1	16.169	0.05597	0
1	16.888	0.063123	0
1	16.942	0.066703	0
1	17.943	0.052598	0
1	19.597	0.026317	0
1	20.115	0.019472	0
1	20.207	0.023866	0
1	20.7	0.016556	0
1	22.021	0.013256	0
1	22.68	0.01025	0
1	23.999	0.006928	0
1	24.025	0.0061189	0
